# Cigarette Cravings, Impulsivity, and the Brain

**DOI:** 10.3389/fpsyt.2015.00125

**Published:** 2015-09-08

**Authors:** Stéphane Potvin, Andràs Tikàsz, Laurence Lê-Anh Dinh-Williams, Josiane Bourque, Adrianna Mendrek

**Affiliations:** ^1^Centre de Recherche de l’Institut Universitaire en Santé Mentale de Montréal, Montreal, QC, Canada; ^2^Department of Psychiatry, Faculty of Medicine, University of Montreal, Montreal, QC, Canada; ^3^Department of Psychology, University of Toronto, Toronto, ON, Canada; ^4^Centre de Recherche de l’Hôpital Sainte-Justine, Montreal, QC, Canada; ^5^Department of Psychology, Bishop’s University, Lennoxville, QC, Canada

**Keywords:** cigarette, cravings, fMRI, individual differences, impulsivity

## Abstract

Craving is a core feature of tobacco use disorder as well as a significant predictor of smoking relapse. Studies have shown that appetitive smoking-related stimuli (e.g., someone smoking) trigger significant cravings in smokers impede their self-control capacities and promote drug seeking behavior. In this review, we begin by an overview of functional magnetic resonance imaging (fMRI) studies investigating the neural correlates of smokers to appetitive smoking cues. The literature reveals a complex and vastly distributed neuronal network underlying smokers’ craving response that recruits regions involved in self-referential processing, planning/regulatory processes, emotional responding, attentional biases, and automatic conducts. We then selectively review important factors contributing to the heterogeneity of results that significantly limit the implications of these findings, namely *between-* (abstinence, smoking expectancies, and self-regulation) and *within-*studies factors (severity of smoking dependence, sex-differences, motivation to quit, and genetic factors). Remarkably, we found that little to no attention has been devoted to examine the influence of personality traits on the neural correlates of cigarette cravings in fMRI studies. Impulsivity has been linked with craving and relapse in substance and tobacco use, which prompted our research team to examine the influence of impulsivity on cigarette cravings in an fMRI study. We found that the influence of impulsivity on cigarette cravings was mediated by fronto-cingulate mechanisms. Given the high prevalence of cigarette smoking in several psychiatric disorders that are characterized by significant levels of impulsivity, we conclude by identifying psychiatric patients as a target population whose tobacco-smoking habits deserve further behavioral and neuro-imaging investigation.

## Introduction

According to recent estimates, there are currently over a billion smokers worldwide and another 300 million are foreseen for 2030 (1). The main issue with these numbers is that smoking harms nearly every organ in the body and is associated with significant disease and mortality (2). Among chronic cigarette smokers, it is estimated that about 50% will eventually be killed by tobacco-related diseases (3). Despite the growing recognition of the harmful health effects of cigarette, many smokers persist in their use and have great difficulty quitting. Although over 70% of smokers want to quit, only 5–17% of quit attempts are successful without proper support (4). For such reasons, tobacco is deemed as one of the most addictive drugs and significant research has been dedicated to the understanding of the psychobiological mechanisms underlying its addictive nature. As craving is a core feature of tobacco use disorder and a significant predictor of smoking relapse (5), several neuro-imaging studies have been performed to elucidate the neural mechanisms underlying smoking motivation. This literature has shown that the brain circuits involved in cigarette craving are not restricted to the classic brain reward system, but also encompass regions involved in self-referential processes and action planning, among others (6). Although we have gained valuable knowledge on the neurophysiology of cigarette cravings, the implications of this literature is limited by the heterogeneity of results. One of the overlooked sources of heterogeneity is impulsivity, a personality trait playing a key role in addiction (7). Recently, our team has published a paper in *Frontiers in Psychiatry* (8), examining the influence of impulsivity on brain activations triggered by cigarette cues in chronic smokers. Putting this work into context is the aim of the current “focused review”.

KEY CONCEPT 1. Cigarette cravings Cravings are defined as persistent urges, thoughts or desires to smoke a cigarette. Considered a core feature of tobacco use disorder in the recent DSM-V, cravings are one of the most consistent predictors of relapse in previous smokers.

KEY CONCEPT 2. Impulsivity Impulsivity is a predisposition toward rapid, unplanned reactions to internal or external stimuli without regard for the negative consequences of these reactions for the impulsive individuals and others.

## Appetitive Smoking Cues

### The clinical relevance of cue reactivity

For many smokers, the most worrying aspects of quitting are the relentless urges or desires to smoke a cigarette. Cravings tend to decrease in strength and frequency with longer abstinence period, yet a minority of ex-smokers still report strong urges 6 months after quitting (9). Empirical studies have shown that cravings – a core feature of tobacco use disorder in the DSM-V – are one of the most consistent predictors of relapse in ex-users (5), and accordingly their reduction is a primary objective of cessation treatment in smokers.

Craving is often defined as a subjective experience of wanting to use a drug, and thereby orienting oneself toward drug taking. Classical conditioning models stipulate that cravings are situation-specific and persistent, such that they can be triggered by stimuli previously associated with drug use, and reinstated years after abstinence (10). Tiffany and Conklin (11) have complemented this model by arguing that craving is a multifaceted construct that involves various cognitive processes, including memory recollection of past drug taking and expectancy of subsequent use. According to them, drug-taking behaviors may occur in the absence of craving because they become automatized in the transition phase to dependence. The role of craving would therefore consist of cognitive processes that can fuel or prevent the execution of automatized drug use habits.

Craving may be divided into tonic (background) and phasic urges for a drug, both of which have been associated with smoking relapse (5, 12, 13). They, respectively, reflect slowly changing states induced by abstinence, and a peak response with fast onset to appetitive drug cues. In experimental settings, exposure to appetitive smoking-related stimuli (e.g., videos, pictures, a lit cigarette) can trigger intense *acute* cravings (14) in abstinent smokers, even following a decline in *tonic* cravings. Likewise, a variety of laboratory stressors can elicit acute cravings in smokers (15). These experimental paradigms offer a clear opportunity to improve our understanding of relapse mechanisms and the inability to quit smoking.

### Neural correlates of smokers’ response to appetitive smoking cues

Over the last decade, considerable efforts have been spent on elucidating the neurobiological bases of cigarette cravings. Most studies used functional magnetic resonance imaging (fMRI) and presented pictures or videos (85% of studies) to depict smoking situations likely to provoke cravings. Relative to neutral material, appetitive smoking-related stimuli have been shown to consistently elicit brain activations throughout cortical and sub-cortical regions in both abstinent and non-abstinent smokers (8, 16–25). A meta-analysis by Engelmann et al. (6) showed that during the viewing of appetitive cigarette-related cues, most significant clusters of activations were located within the extended visual cortex, followed by the anterior and posterior cingulate gyri, the medial and dorso-lateral prefrontal cortex, and the superior and middle temporal gyri. Smaller clusters were also observed in the insula and (dorsal) striatum. The widespread brain reactivity to drug cues validates the notion that craving is a multidimensional phenomenon implicating several cognitive processes. Contrary to the postulates of classic theories of drug addiction (10), these findings reveal that the brain craving response is not confined to the brain reward circuitry, and that other key brain regions need to be recruited in order to fully experience the complex craving response. The available results highlight a vastly distributed neuronal network underlying smokers’ cravings, which stresses the importance of attentional/visual processing (extended visual system) (16, 22, 24), self-referential processing [precuneus, posterior cingulate cortex (PCC)] (26–28), planning/regulatory processes [anterior cingulate cortex (ACC) and dorso-lateral prefrontal cortex (dlPFC)] (17, 29), emotional/interoceptive processes (insula) (18, 19, 29–31), and learning of automatic conducts (dorsal striatum) (28, 31, 32). Among these regions, frontal and limbic (insula) sub-regions seem to be the most directly related to the subjective experience of cigarette cravings (33).

In addition to the vast neuro-imaging literature examining brain responses of smokers to appetitive smoking-related material, a few authors have investigated the specific neural correlates of appetitive drug-related processing by comparing it to the processing of appetitive stimuli other than drugs. As hypothesized by Volkow et al. (34), drug addicts displayed a significant decrease in incentive salience toward natural compared to drug rewards, which helps explaining the heightened motivation for drug use observed in this population. Specifically in nicotine dependence, chronic cigarette smokers were found to be more reactive to cigarette cues relative to erotic pictures, whereas the opposite was observed in non-smokers. This difference in brain reactivity between smokers and non-smokers was found in the middle frontal gyrus (Brodmann area 6/8) (35), a region near the supplementary motor area thought to be involved in action planning and expectancy. This suggests a greater orientation, in smokers, toward the appetitive value of drugs than that of natural rewards. Finally, Versace et al. (36) found that smokers having decreased activity in the dorsal striatum while viewing erotic and romantic pictures compared to smoking cues were significantly more likely to have relapsed 6 months after quitting. This suggests that smokers with decreased dorsal striatum activity to pleasant stimuli have greater difficulty quitting. It also suggests that reinforced drug habits promote a biased sensitivity toward the appetitive value of smoking compared to that of other rewards.

Our team has further explored the specificity of the neural correlates of appetitive drug-related processing by contrasting the brain responses of smokers to appetitive and aversive smoking cues (37). The rationale of this study was based on evidence showing that smokers are roused by appetitive smoking stimuli, and that their consumption tends to be only mildly affected by anti-smoking stimuli depicting smoking’s negative value (38, 39). Using fMRI, 30 chronic smokers viewed appetitive smoking-related, aversive smoking-related, and neutral images. Appetitive smoking-related images elicited increased activations in the medial prefrontal cortex (mPFC), the PCC and the precuneus, compared to aversive smoking-related cues. This study demonstrated that smokers recruit brain regions involved in the processing of self-relevant material in response to appetitive smoking cues, compared to anti-smoking stimuli, highlighting a neurophysiologic bias toward the positive, rather than negative, value of smoking.

## Sources of Heterogeneity

When looking at individual studies assessing the neural correlates of cigarette craving, one finds that several factors contribute to the heterogeneity of findings *between* studies, such as abstinence levels, smoking expectancy, and craving suppression. There is also high individual variability *within* studies in the brain activations associated with exposure to smoking cues. It is therefore crucial to further understand this variability in brain reactivity to appetitive cigarette stimuli. Smoking dependence severity, sex-differences, motivation to quit, and genetic factors have been studied as potential factors of inter-individual variability in cigarette craving-induced brain responses. Comparatively, little attention has been paid to anxiety, depression, and personality traits. Table 1 summarizes the list of factors influencing brain reactivity to appetitive smoking cues.

**Table 1:** **Factors influencing brain reactivity to appetitive smoking cues**.

Variable	Observations	Relevant reference
**Between-study factors**		
Abstinence	During abstinence, cue-elicited cravings are more intense, and elicit stronger activations in the rostral anterior cingulate gyrus	(40)
Expectancy	Smokers display greater rostral prefrontal cortex activations when they are told that they will be allowed to smoke a cigarette immediately after the scan	(22, 27, 32, 41)
Self-regulation of cravings	Smokers display increased recruitment of frontal and cingulate regions when they are actively seeking to resist cigarette cravings	(16, 26, 42, 43)
**Within-study factors**		
Smoking dependence severity	Smoking dependence severity is positively associated with increased brain reactivity to appetitive smoking cues. The brain regions underlying this increased brain reactivity remain to be determined. Heterogeneity of results is a concern	(19, 23, 31, 44–46)
Sex-differences	Preliminary results that need to be confirmed suggest that the brain reactivity to appetitive smoking cues in women smokers is increased during the follicular phases of the menstrual cycle	(21, 47)
Motivation to quit	Preliminary evidence suggests that motivation influences the brain responses of smokers to appetitive smoking cues	(48, 49)
Genetic factors	Preliminary studies have linked dopamine- and acetylcholine-related genetic polymorphisms to the brain reactivity of smokers to appetitive smoking cues	(18, 19, 30)
Impulsivity trait	Impulsivity trait is associated with increased cue-elicited cigarette cravings. This relationship may be mediated by fronto-cingulate mechanisms	(8)

### Between-study heterogeneity

Of all the variables that may influence the neural correlates of cue-elicited cigarette cravings, abstinence has been the most studied. On theoretical grounds, Wilson and Sayette (40) proposed that *moderate* and *uncontrollable* cravings may trigger substantially different brain responses. Consistently with this idea, the meta-analysis from Engelmann et al. (6) found that studies performed in deprived smokers (who report more intense cravings) had increased activations in the right superior frontal and the left lingual gyrus, relative to studies performed in non-deprived smokers. However, this meta-analysis also showed sizeable overlap between both sets of studies, which produced activations in 44 widespread clusters (6). Recently, Wilson and Sayette (40) updated the meta-analysis of Engelmann et al. (6). By contrasting 24 functional imaging studies involving deprived and non-deprived smokers, this updated meta-analysis showed that deprived smokers elicit stronger activations of the rostral ACC than smokers allowed to smoke *ad libitum* before the scanning session. Interestingly, the rostral ACC is thought to play a critical role in the neurobiology of addiction, given that it receives dopaminergic inputs from the ventral tegmental area (10) and is involved in self-referential processes (50).

Smoking expectancy has also been shown to influence brain reactivity to appetitive smoking cues in chronic smokers. Wilson et al. (22) found that the expectation of being allowed to smoke a cigarette immediately after the scanning session was associated with increased activations in the ventro-medial PFC, the precentral gyrus, and the right middle temporal gyrus. The same research team subsequently confirmed the influence of smoking on rostral PFC activations, but only in smokers unmotivated to quit (32). Hayashi et al. (41) found that smoking expectancy is associated with increased activation in the dlPFC. Finally, McBride et al. (27) found an association between smoking expectancy and activations in several frontal regions (dlPFC, dACC, dmPFC, mOFC), the PCC, and the precuneus. Overall, these results suggest that smoking expectancy is associated with increased activations in regions involved in action planning and reward valuation.

Finally, the ability to resist cigarette cravings was studied in four fMRI studies. Brody et al. (16) found that the PCC was engaged when participants were actively trying to suppress their urges, but not when they craved without trying to resist. Hartwell et al. (26) found that the ability to resist cigarette cravings was associated with increased activations in the left ACC, the ventro-/mPFC and the dlPFC. Zhao et al. (42) found that the inhibition of cue-induced cigarette cravings (via re-appraisal) was associated with increased activations of the right dorsal ACC. Finally, Kober et al. (43) showed that cognitive down-regulation of cigarette cravings was associated with increased activations in regions involved in cognitive control [e.g., the dlPFC, dmPFC, and ventro-lateral prefrontal cortex (vlPFC)]. It is therefore possible that smokers with poor self-control abilities experience greater difficulty in controlling their urges, and this is (partially) mediated by lower activation of fronto-cingulate regions. Such results highlight the importance of self-regulation abilities in the subjective experience of cigarette cravings.

### Within-study heterogeneity

Attention has also been paid to the severity of smoking dependence and how it may influence brain cue reactivity. Apart from Vollstadt-Klein et al.’s (23) study, which found that severe smoking dependence was associated with *decreased* activations in the amygdala, hippocampus, putamen, and thalamus, most studies have shown that smoking dependence is *positively* associated with smoking cue-elicited brain activations. Thus far, severe smoking dependence has been associated with *increased* activations in craving-related frontal and temporal regions (44, 45), the insula (19, 44), and the (superior) parietal cortex (31, 45, 46). As such, these results suggest that cigarette cues provoke a stronger brain craving response in smokers whose dependence is more severe.

KEY CONCEPT 3. Brain cue reactivity Relative to neutral stimuli, appetitive smoking-related cues have been shown to elicit significant activations within the extended visual cortex, the (medial and dorsolateral) prefrontal cortex, the (anterior and posterior) cingulate, the temporal cortex, the insula, and the dorsal striatum. The widespread brain reactivity to cigarette cues suggests that craving is a multidimensional construct.

In view of the clinical evidence showing that women and girls take less time to become dependent after initial use and have more difficulties quitting the habit (51, 52), preliminary studies examined the influence of sex-differences on the neural correlates of cigarette cravings. One of the factors contributing to these differences may be that women crave cigarettes more than men and that their desire to smoke is influenced by hormonal fluctuations across the menstrual cycle. Therefore, we performed a study involving tobacco-smoking men (*n* = 15) and women (*n* = 19) who underwent an fMRI session, during which neutral and smoking-related images, known to elicit craving, were presented (47). Women were tested twice; once during early follicular and once during mid-luteal phase of their menstrual cycle. The analysis did not reveal any significant sex differences in the cerebral activations associated with craving. This result contrasts with the fMRI findings from McClernon et al. (21), who found that women smokers exhibited larger cue reactivity in the right putamen, bilateral cuneus, and left middle temporal gyrus, while men had greater responses in the left hippocampus and left orbitofrontal cortex. Nevertheless, our study showed that brain activations in women varied across their menstrual cycle. More precisely, we found that female smokers had increased activations in the right angular/middle temporal gyrus in the follicular phase compared to the luteal phase. This latter result echoed the clinical reports of fluctuations in tobacco intake, withdrawal symptoms, and relapse rates across the menstrual cycle in women smokers (53, 54), as well as the preclinical findings showing that higher levels of estrogen (follicular phase) are associated with more reinforcing effects of addictive drugs, whereas higher levels of progesterone (luteal phase) are associated with less reinforcing effects (55).

Some authors have shown that one’s intention to quit can impact prefrontal and limbic activity toward cigarette pictures (48) Furthermore, it was shown that smokers who are dissatisfied with their smoking behavior, relative to those more accepting of their tobacco use, were more reactive (e.g., orbitofrontal and limbic activations) to appetitive cigarette cues (49).

Finally, genetic factors have also been shown to modulate brain reactivity to smoking cues. An association has been found between the dopamine transporter gene SLC6A3 polymorphism and ventral striatal and medial orbitofrontal activations in response to smoking cues (18), a finding that was subsequently replicated in another independent sample (19). In addition, an association has been observed between the nicotinic receptor alpha-5 subunit gene (rs16969968) polymorphism and smoking cue-elicited activations in the hippocampus and dorsal striatum (30).

## Impulsivity

Personality traits, such as impulsivity, have been largely overlooked as potential factors contributing to heterogeneity in the fMRI studies on cigarette cravings. Impulsivity constitutes a key diagnostic criterion for several mental disorders, most importantly for antisocial personality disorder, borderline personality disorder, impulse-control disorders, and attention deficit and hyperactivity disorder (ADHD) (56, 57). Yet, the definition and dimensions of impulsivity remain a source of debate. The most widely accepted definition of impulsivity is a predisposition toward rapid, unplanned reactions to internal or external stimuli without regard for the negative consequences of these reactions for the impulsive individuals and others (58). There are (at least) five dimensions of impulsivity, namely: (i) the difficulty in maintaining one’s attention toward stimuli; (ii) the initiation of actions without forethought or planification; (iii) sensation/novelty seeking; (iv) the indifference toward the long-term consequences of one’s choices and actions; and (v) the preference of immediate over larger delayed rewards, regardless of its disadvantages (59–63). Several scales have been developed to measure the different components of impulsivity (60, 63, 64). Research using these scales has shown that impulsivity has the characteristics of a personality trait: it is relatively stable in time and genetically transmitted (65, 66).

Impulsivity has been extensively studied using cognitive tasks. Bari and Robbins (67) have recently proposed a model that defines two dimensions of impulsivity largely studied in cognitive science: (1) a *rapid action form*, which refers to behaviors that are performed without consideration for their consequences and (2) a *slower form* involving the adoption of desired behaviors despite their negative consequences [Note: other researchers have proposed similar models (68–70)]. Whereas the rapid form involves cognitive control processes allowing the flexible adaptation of behaviors to meet current demands (71, 72), the slower form entails value-based decision-making processes, which enable the selection of a behavior based on predicted positive or negative outcomes (73). While the rapid action form of impulsivity is assessed using response inhibition paradigms, such as the Go/No-Go task, which require the suppression of a pre-potent motor response (71, 74, 75), the slower form of impulsivity is assessed using risky decision-making tasks that include rewards, such as the *Iowa Gambling task* (76, 77). Other components of impulsivity, such as the ability to delay rewards (78–82), have been studied in cognitive science, but to a lesser extent. Moreover, delay-discounting tasks have been used less frequently than response inhibition and decision-making tasks in the functional imaging studies performed on the neural bases of impulsivity.

### The neurobiology of impulsivity

Response inhibition and risky decision-making tasks have been largely employed to examine the neural bases of impulsivity, using functional imaging. Although there may be a partial overlap between the neural mechanisms underlying cognitive control and risky decision-making, the available evidence suggests that response inhibition tasks recruit a fronto-lateral executive network (74, 75, 83–85), while risky decision-making tasks recruit reward-related structures (86–88). Response inhibition tasks require withholding an already selected or initiated motor response. A recent meta-analysis of 30 fMRI studies (89) has shown that the vlPFC, the dlPFC, and the (dorsal) ACC are critically involved in response inhibition, presumably exerting executive control over the motor response. On the other hand, risky decision-making relies on mental processes that are mostly driven by reward seeking. A large-scale meta-analysis of 206 fMRI studies (90) has shown that rewards activate a valuation system composed of the ventro-medial PFC, the ventral ACC, and the (ventral) striatum. This is consistent with recent fMRI studies, highlighting the crucial role of these regions in risky decision-making (77, 91, 92). Thus, mounting evidence suggests that cognitive control involves fronto-lateral mechanisms (83), while risky decision-making relies on reward-related structures (70, 93).

### Impulsivity and cigarette smoking

Impulsivity plays a critical role in the etiology of substance use disorders (94). In adolescents, impulsivity has repeatedly been shown to predict the onset of substance use and subsequent escalation of use (95, 96). In the case of tobacco, longitudinal studies have shown that impulsivity/hyperactivity traits at 12 years old predicted cigarette smoking 2 years later (97). In adolescents, it has also been shown that the preference for immediate rewards over larger delayed rewards promoted smoking acquisition (96). Likewise, an epidemiological study from Norway showed that impulsivity, as measured with the *Barratt Impulsiveness Scale* (BIS), was significantly associated with cigarette smoking initiation, even after controlling for education level (98).

Increased impulsivity levels have repeatedly been highlighted in chronic cigarette smokers, relative to non-smokers, using various clinical and cognitive instruments. Using the BIS, several studies have reported high levels of impulsivity traits in chronic cigarette smokers, relative to non-smokers (78, 81, 99–101). In addition, it has been shown, using the Go–No-go task, that compared to healthy volunteers, cigarette smokers fail more frequently to inhibit pre-potent motor responses (82, 99). Finally, it has been repeatedly demonstrated that cigarette smokers are less likely than non-smokers to choose large delayed rewards over small immediate ones in delay-discounting tasks (78–82). On neurobiological grounds, preliminary fMRI studies have shown that chronic smoking is associated with decreased ventral striatal activations during reward-related tasks (102, 103), and abnormal lateral prefrontal activations during the Stroop task (104).

In abstinent smokers, impulsivity has been shown to be a significant predictor of smoking relapse. In both adolescent and adult smokers receiving treatment for smoking cessation, smokers with impulsivity traits were found to be less likely to remain abstinent compared to non-impulsive smokers (105–107). Importantly, smokers who remained abstinent and those who relapsed did not differ at baseline in terms of number of smoked cigarettes, motivation to quit, confidence in their own abilities to quit, age, and sex. Similarly, Doran et al. (108) have shown, among smokers who relapsed during a 1-month intervention for smoking cessation, an association between impulsivity and shorter time to relapse. In order to explain the nature of the association between impulsivity and relapse, VanderVeen et al. (109, 110) hypothesized that during abstinence, the rewarding value of tobacco may be increased in impulsive smokers; that is, impulsive smokers may crave more for cigarettes during abstinence than non-impulsive ones.

### Impulsivity and cigarette cravings

A growing number of studies have evidenced an association between impulsivity trait and cravings for alcohol (111–113), cocaine (114, 115), methamphetamine (115), and tobacco (116–118). Although the link between impulsivity and craving is increasingly substantiated by evidence, the mechanisms underlying this association remain poorly understood. Surprisingly, the neural mechanisms mediating the influence of impulsivity on cigarette cravings had not been studied (to our knowledge) until our research team sought to do so.

### Impulsivity and the neural correlates of cigarette cravings

Impulsivity plays a pivotal role in the substance use onset, substance use relapse, and craving for psycho-active substances, including tobacco. Recently, our team performed an fMRI study seeking to examine the influence of trait impulsivity on the neural correlates of cue-elicited cigarette cravings (8). Thirty to 40 min prior to the scanning session, participants smoked a cigarette. While in the scanner, 31 chronic smokers viewed appetitive smoking-related and neutral images and reported their subjective craving levels. They also completed the BIS. The processing of appetitive smoking cues elicited, in smokers, activations in midline brain regions (the mPFC, ACC, and PCC) that have been repeatedly found to be activated in fMRI studies on tobacco cravings (6), and to be involved in self-referential processing (119–121). In addition, we observed a significant positive relationship between the total BIS score and subjective craving levels (*r* = 0.624; *p* < 0.001). Among second-order factors of the BIS (attentional, motor, and non-planning), it was the non-planning subscale that was the most significantly correlated with craving ratings (*r* = 0.625; *p* < 0.001). A negative correlation (*r* = −0.449; *p* = 0.015) was also observed between the total BIS score and activations in the PCC. Among second-order factors, only the non-planning subscale was significantly correlated with PCC activations (*r* = −0.440; *p* = 0.017). Such results were consistent with previous findings showing that the PCC is involved in resisting cigarette cravings (16). Based on the observed associations between impulsivity and PCC activations, we conducted functional connectivity analyses with the psycho-physiological interaction (PPI) method, using the PCC as the seed region. PPI analyses revealed significant negative coupling between the PCC and the dorsal ACC, the right dlPFC, and a region in the vicinity of the left insula. Thus, lower activations of the PCC, found in impulsive smokers, were associated with higher activations of brain regions involved in emotional (insula) and attentional (dACC and dlPFC) responses to smoking cues. Our findings highlighted the need for further investigations on the role of the PCC in drug addiction, as it is one of the most consistently activated regions in fMRI studies examining the neural correlates of cue-induced alcohol, drug, and tobacco cravings (6, 122, 123). Besides its role in self-referential processing, the PCC may be involved in the ability to resist cravings for psycho-active substances, including tobacco. Theoretically, an impaired functioning of the PCC may (indirectly) result in a lack of self-control over substance (tobacco) cravings. Although the PCC is not assumed to be a core cognitive control region, a neuro-imaging meta-analysis of 24 fMRI studies using stop-signal tasks recently showed that the PCC is one of the main activated regions during response inhibition (124).

Contrary to our expectations, we did not observe any *direct* relationship between impulsivity and dysfunctional activations in the dlPFC or the vlPFC (though the dlPFC emerged as significantly coupled with the PCC). Critically involved in cognitive control (74, 75, 89), these frontal regions have been shown to be implicated in efforts to inhibit cigarette cravings (26, 43). Although the reasons for these negative findings remain elusive, it is possible that the impulsivity *trait* refers to more complex neural processes than those involved in decision-making and cognitive control. Highly impulsive individuals are stimulus-bound and focus on immediate rather than long-term events. They also tend to lack introspection and have impaired mentalization abilities (125). Such characteristics are very unlikely to be captured by risky decision-making or response inhibition tasks. Over and above the dlPFC and vlPFC, the PCC is critical for self-relevant processes, such as mindfulness, mentalizing, and self-reflection (119–121). Altogether, these results should encourage researchers who will pursue future studies on the neurobiology of impulsivity in the addiction field to pay attention to components of impulsivity other than cognitive control and risky decision-making.

## Future Perspectives: Tobacco Smoking in Psychiatric Disorders

Several psychiatric disorders are characterized by high or moderate levels of impulsivity, namely ADHD, bipolar, cluster-B personality, psychotic, and substance use disorders (7, 58, 126–129). Moreover, the prevalence of cigarette smoking is increased in patients with psychiatric disorders. In the US, approximately 29% of the population has a psychiatric disorder, and these individuals consume approximately 41% of the production of tobacco in the country (130). Although the prevalence of tobacco smoking has declined in the general population during the last decades, the consumption of tobacco products remained strikingly elevated in patients with schizophrenia, bipolar disorder, major depressive disorder, post-traumatic stress disorder (PTSD), and ADHD, with prevalence estimates of 60–74, 66, 57, 40–86, and 40–42%, respectively (130–136). Elevated rates of tobacco smoking have also been observed in individuals with substance use disorders (137). It has been shown, indeed, that more than a third of patients with an alcohol use disorder and more than half of individuals who misuse (abuse/dependence) illicit drugs are also nicotine dependent (138). Therefore, psychiatric patients represent populations of interest for the study of the relationship between impulsivity and tobacco cravings, at both the behavioral and neural level.

### Explanatory models

Two major hypotheses have been advanced to account for the elevated prevalence of tobacco smoking in psychiatric patients. The *self-medication hypothesis* proposes that psychiatric patients smoke cigarettes to relieve their psychiatric symptoms (anxiety, depression) or cognitive deficits (139). In support of this hypothesis, numerous studies have shown that the acute administration of nicotine (the main psycho-active agent of tobacco) to schizophrenia patients improves some of their cognitive deficits (e.g., attention, speed of processing, and working memory), and their impaired ability to filter out irrelevant information (140–142). Preliminary studies have shown that nicotine administration (or tobacco smoking) also improves cognitive performance in patients with ADHD (143, 144), bipolar disorder (145), and depression (20, 145).

Despite these findings, the self-medication hypothesis has been criticized over the years for its implied justification of tobacco smoking in psychiatric patients. Alternatively, the *addiction vulnerability hypothesis* proposes that neurobiological dysfunctions of the brain reward system, common to psychiatric and substance use disorders, make psychiatric patients more vulnerable to the rewarding effects of various psycho-active substances, including tobacco (139, 146, 147). In support of the *addiction vulnerability hypothesis*, several studies have shown that tobacco is more reinforcing for schizophrenia patients than it is for non-psychiatric smokers. Schizophrenia patients are more prone to smoke high-tar cigarettes, and they tend to smoke more cigarettes per day, extract more nicotine per cigarette, and have higher serum nicotine and cotinine (nicotine metabolite) levels compared to control smokers (148, 149). Preliminary evidence also suggests that cravings for cigarette are increased in schizophrenia patients (150, 151), 15 min or 72 h after smoking their last cigarette. Preliminary studies have shown that cigarette cravings are also elevated in other psychiatric disorders, including ADHD and PTSD (152–155). Despite these evidences, the neural correlates of tobacco cravings have been scarcely examined in psychiatric patients or individuals with psychiatric vulnerabilities (156–158).

Future studies will need to replicate and extend the findings demonstrating that cigarette cravings are increased in psychiatric patients, and to clarify whether bottom-up (e.g., motivational salience) or top-down (e.g., self-regulation) neural mechanisms explain these findings. To explore this self-regulation hypothesis, impulsivity will need to be measured in these populations. Although most studies in the comorbidity field have not focused on tobacco smoking specifically, mounting evidence shows that impulsivity is a key mediator of the association between substance use and psychiatric disorders, including ADHD, bipolar disorder, cluster-B personality disorders, and schizophrenia (7, 126–128). Several fMRI studies have examined the neural correlates of impulsivity in psychiatric patients (mostly bipolar disorder and schizophrenia) having no comorbid substance use disorders. Using various response inhibition tasks, these studies have shown that psychiatric patients have abnormal brain activations in regions involved in cognitive control, such as the dlPFC, the vlPFC, and/or the cingulate gyrus (159–163). Despite the variability in results, such findings pave the way to future neuro-imaging studies on drug (cigarette) cravings in psychiatric patients examining the interactions between cognitive control brain regions and those involved in craving experience.

KEY CONCEPT 4. Comorbidity The prevalence of cigarette smoking is elevated in several psychiatric disorders characterized by high or moderate levels of impulsivity, namely attention deficit hyperactivity disorder, bipolar disorder, cluster-B personality disorders, psychotic disorders, and substance use disorders.

## Conclusion

Given that craving is a core feature of tobacco use disorder and a significant predictor of smoking relapse, several fMRI studies have examined the neural correlates of (cue-induced) cigarette urges. The available literature has demonstrated that cigarette cravings elicit activations in regions involved in self-referential processing, planning/regulatory processes, emotional responding, attentional biases, and automatic conducts. Unfortunately, the implications of this literature are limited by the heterogeneity of results. Sources of heterogeneity include both methodological (abstinence, smoking expectancies, self-regulation) and individual factors (severity of smoking dependence, sex-differences, motivation to quit, genetic factors). Surprisingly, the potential influence of impulsivity has been largely ignored, although impulsivity is positively associated with cigarette cravings and smoking relapse. The influence of impulsivity on cigarette cravings is possibly mediated by fronto-cingulate mechanisms, as recently demonstrated by our team. Future behavioral and neuro-imaging studies will need to pay attention to the high prevalence of cigarette smoking in several psychiatric disorders, as these disorders are characterized by significant impulsivity.

## Conflict of Interest Statement

The authors declare that the research was conducted in the absence of any commercial or financial relationships that could be construed as a potential conflict of interest.
